# Enhancing Techniques for Determining Inflammatory Edema Formation and Neutrophil Accumulation in Murine Skin

**DOI:** 10.1016/j.xjidi.2022.100154

**Published:** 2022-09-07

**Authors:** Ali A. Zarban, Hiba Chaudhry, Davide Maselli, Xenia Kodji, Joao de Sousa Valente, Justin Joachim, Silvia Cellone Trevelin, Johannes van Baardewijk, Fulye Argunhan, Aleksandar Ivetic, Manasi Nandi, Susan D. Brain

**Affiliations:** 1 Section of Vascular Biology and Inflammation, British Heart Foundation Centre of Research Excellence, School of Cardiovascular and Metabolic Medicine & Sciences, King's College London, London, United Kingdom; 2 Department of Pharmacology and Toxicology, College of Pharmacy, Jazan University, Jazan, Saudi Arabia; 3 James Black Centre, British Heart Foundation Centre of Research Excellence, School of Cardiovascular and Metabolic Medicine & Sciences, King's College London, London, United Kingdom; 4 Agency for Science, Technology and Research (A^∗^STAR) - Skin Research Institute of Singapore (SRIS), Singapore, Singapore; 5 Ray Global Education, Shanghai, China; 6 School of Cancer & Pharmaceutical Sciences, Faculty of Life Sciences & Medicine, King's College London, London, United Kingdom

**Keywords:** CGRP, calcitonin-gene related peptide, EB, Evans blue, i.d., intradermally, i.v., intravenously, MPO, myeloperoxidase, OD, optical density, SP, substance P, TMB, 3,3′,5,5′-tetramethylbenzidine

## Abstract

Inflammatory edema formation and polymorphonuclear leukocyte (neutrophil) accumulation are common components of cutaneous vascular inflammation, and their assessment is a powerful investigative and drug development tool but typically requires independent cohorts of animals to assess each. We have established the use of a mathematical formula to estimate the ellipsoidal-shaped volume of the edematous wheal or bleb after intradermal injections of substances in mice pretreated intravenously with Evans blue dye (which binds to plasma albumin) to act as an edema marker. Whereas previous extraction of Evans blue dye with formamide is suitable for all strains of mice, we report this quicker and more reliable assessment of edema volume in situ. This therefore allows neutrophil accumulation to be assessed from the same mouse using the myeloperoxidase assay. Importantly, we examined the influence of Evans blue dye on the spectrometry readout at the wavelength at which myeloperoxidase activity is measured. The results indicate that it is feasible to quantify edema formation and neutrophil accumulation in the same mouse skin site. Thus, we show techniques that can assess edema formation and neutrophil accumulation at the same site in the same mouse, allowing paired measurements and reducing the total use of mice by 50%.

## Introduction

Inflammatory edema formation is a hallmark of inflammation and a consequence of increased microvascular permeability that leads to plasma extravasation (Park-Windhol and D'Amore, 2016). There are several definitions of the phenomenon such as wheal or induration (used for skin) as well as vascular leak. Indeed, the phenomenon is associated with hyperinflammation in a wide range of skin conditions, in addition to in other organs (Nagy et al., 2012). The acute increased microvascular permeability is considered to occur in postcapillary venules (Majno et al., 1967, 1961) through two classic mechanisms: first, through mediators such as histamine and substance P (SP) that act through endothelial receptors to stimulate increased microvascular permeability (Brain and Williams, 1985; Cao et al., 1999) and second, through a leukocyte (generally considered neutrophil-dependent)-increased microvascular permeability (Wedmore and Williams, 1981). The concept of neutrophil-dependent‒increased microvascular permeability is not fully understood (DiStasi and Ley, 2009), with some mediators (e.g., TNF-α) needing to stimulate the upregulation of endothelial receptors before neutrophil interactions and observation of edema formation (Norman et al., 2005). It is essential to maintain endothelial integrity and tight junctions for a healthy vasculature, and therefore, the study of edema formation is important. Acute edema formation may be considered protective, (e.g., insect bites). However, in diseases (e.g., diabetes or acute respiratory distress syndrome), the passage of inflammatory cells and macromolecular proteins into inflamed tissues can lead to serious complications (Rask-Madsen and King, 2013; Rizzo et al., 2015). Edema formation and neutrophil accumulation are key inflammatory components that are involved in inflammation. Inflammation, depending on the circumstances, can include a large array of mediator pathways and cellular involvement other than neutrophils. In this study, we concentrate on edema formation and neutrophil accumulation and their measurement in the same skin samples.

The skin assay technique to measure inflammatory edema formation was first developed to study histamine-induced hyperpermeability in laboratory species (Miles and Miles, 1952). From there, many adaptations have been made to the assay, including enabling the use in mice. Pontamine blue was replaced by Evans blue (EB) dye that binds noncovalently to plasma albumin (Sawyer et al., 2011). Furthermore, radio-labeled ^125^I-labeled BSA (intravenous) instead of dye has been used to calculate volume (Cao et al., 1999) but is now very difficult to obtain. We present the use of a mathematical equation that takes account of the ellipsoid shape of the wheal, visualized by the extravascular accumulation of intravenously (i.v.) injected EB dye. Owing to binding to plasma albumin, EB acts as a marker of the extravascular accumulation of plasma and therefore of edema formation. This means that samples are kept intact for further investigation, such as for the measurement of neutrophil accumulation at the skin site. Despite being a carcinogen, EB is commonly described as an inert tracer and used historically to measure plasma volume (Evans and Schulemann, 1914). Recently, it has been in drug discovery programs without obvious toxicity (Wang and Lai, 2014).

The use of an equation to calculate edema volume is only feasible if the response can be clearly observed, as in white-furred strains of mice. Dark-skinned mice, on which many genetically modified strains are bred, may be too pigmented. Thus, as a consequence, inflammatory edema formation has often been quantified through formamide extraction of EB dye (Brash et al., 2018; Radu and Chernoff, 2013). This is despite reports on the turbidity of some formamide extracts from certain tissues affecting specificity (Warnick et al., 1995).

We have evaluated the use of the mathematical formula by comparing results through this and the formamide extraction assay. Myeloperoxidase (MPO), an enzyme highly expressed in neutrophils, is established as a biomarker for tissue neutrophil content (Bradley et al., 1982). The edema assay and MPO assay are not traditionally carried out on the same mouse. This is partly because the skin site cannot be further used after formamide extraction. Second, if there is a visual assessment of EB dye, there is an assumption that this will interfere with the assay of MPO activity, which involves optical density (OD) readings in the blue range. This limitation was first reported in 1985, although no data were shown (Mullane et al., 1985). It has been suggested that EB does not interfere with MPO assay (Fliss and Gattinger, 1996; Griswold et al., 1989), but little evidence has been provided.

In this study, we provide evidence that inflammatory edema formation in mouse skin can be measured using a mathematical formula through direct measurements and calculation of the ellipsoid wheal volume. Importantly, the use of the formula is quicker and preserves the skin site for use in other assays, unlike in the formamide extraction technique. Results indicate that through the use of an MPO assay, neutrophil accumulation can be measured in these same skin sites; this is of direct relevance to the reduction of experimental mouse numbers used.

## Results

### Using a mathematical formula for the assessment of edema formation

**Figure 1:**
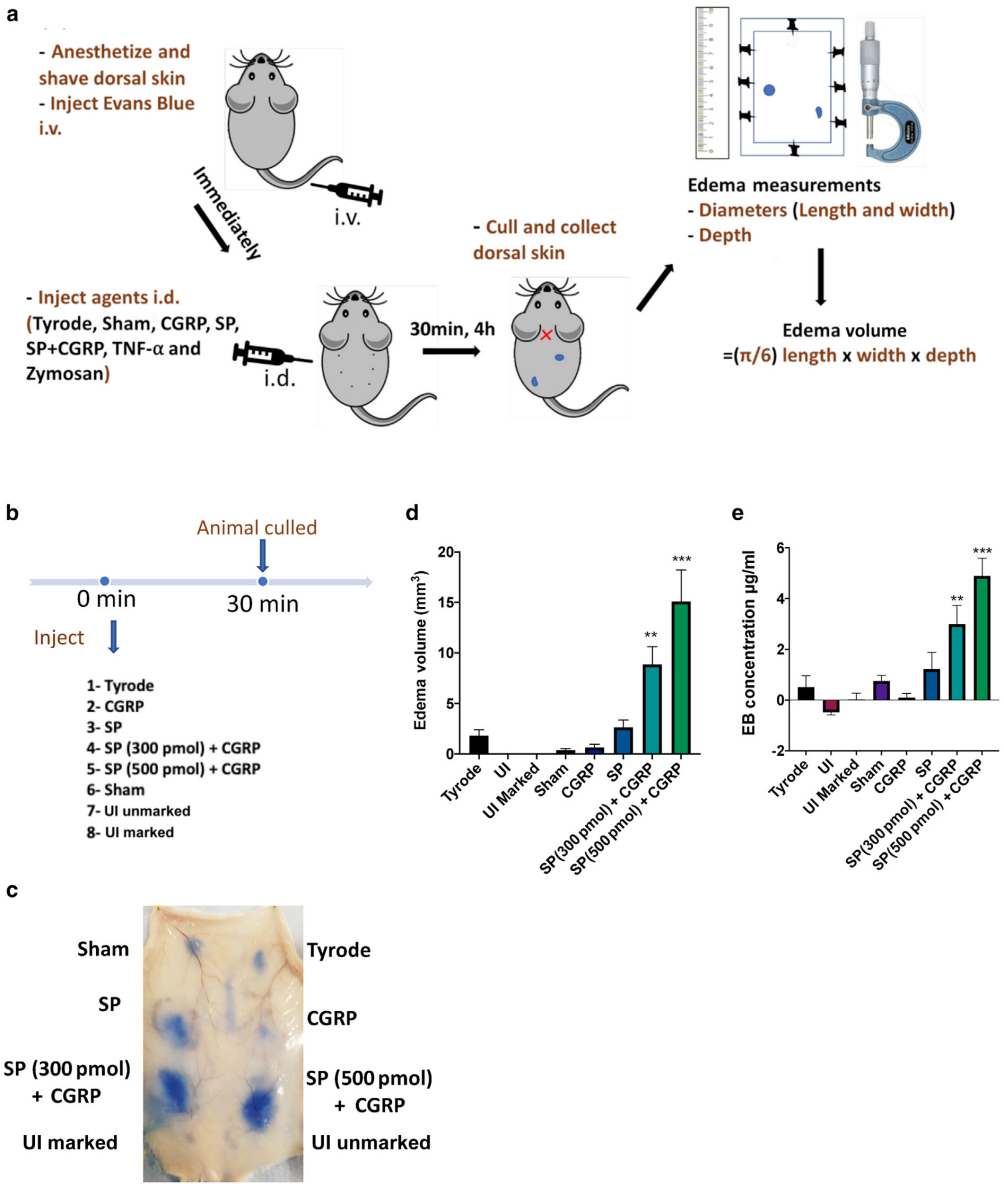
**Using the formula for edema formation**. (**a**) A schematic depiction of the skin assay protocol showing i.v. injection of EB, followed by i.d. injection (50 μl) of test agents, accumulation period, and analysis of sites. (**b**) Timeline for the experiment where the following agents were tested 5 min after i.v. injection of EB: SP (300 pmol) + CGRP (20 pmol), SP (500 pmol) + CGRP (20 pmol), Tyrode, Sham, CGRP (20 pmol), SP (300 pmol), and uninjected sites (marked and unmarked) were evaluated. (**c, d**) Representative results were analyzed by the mathematical formula and (**e**) using the formamide extraction assay. Results are shown as mean ± SEM (n = 5). One-way ANOVA with Dunnett’s post hoc test was performed. ∗∗*P* < 0.01 and ∗∗∗*P* < 0.001 compared with vehicle control (Tyrode’s solution). EB, Evans blue; h, hour; i.d., intradermal; i.v., intravenous; min, minute; SP, substance P; UI, uninjected.

We selected the prolate ellipsoid formula: (π/6) × length × width × depth*,* to estimate the ellipsoidal-shaped volume after studying the shape of the wheals in mouse dorsal skin and accounting for the ability to measure by either rulers or calipers (Figure 1a). To examine for validity, we measured the response to SP (Sigma-Aldrich, St. Louis, MO) when injected with the vasodilator peptide calcitonin-gene related peptide (CGRP) (AnaSpec, Fremont, CA) intradermally (i.d.) at known edema-inducing dose combinations (Cao et al., 1999). The experimental plan included several types of controls: uninjected, uninjected but marked (where the injection site is marked with a black marker), a sham site (needle only, no liquid), and a vehicle control site (Tyrode’s solution, 50 μl i.d.) into the dorsal skin. Immediately before intradermal injections, the mouse received the EB dye (i.v.) (Sigma-Aldrich, St. Louis, MO). After the intradermal injections, edema formation was allowed for 30 minutes (Figure 1b). The study was terminated, and the dorsal skin was dissected and placed vascular side up (Figure 1c). For response measurements, the length and width measurements were taken with a ruler, and after unpinning, the depth was measured using an engineering caliper.

### Validating the use of the formula with the formamide extraction technique

The use of the formula depends on the clear visualization of the edema formed on the mouse dorsal skin. This is not an issue in pale fur strains (e.g., CD1) but becomes problematic in some mice with highly pigmented skin (e.g., certain C57BL/6 mice). To evaluate the validity of the formula, we compared the EB skin assay using the equation from the mathematical formula with EB extraction using the formamide (Sigma-Aldrich, St. Louis, MO) technique in CD1 mice over a 30-minute accumulation period, as shown in Figure 1d and e.

Figure 1d shows the results from the experiment (designed in Figure 1b) using the equation (described in Figure 1a). It is noted that neither SP nor CGRP (when injected alone) induced significant edema formation at the doses used in this experiment. The simultaneous administration of both leads to CGRP potentiating the net edema formation because of the increased blood flow at the site of injection (Brain and Williams, 1985; Cao et al., 1999). Thus, a dose-dependent significant edema formation in response to SP mixed with CGRP is shown (Figure 1d). The higher concentration of SP was introduced to examine the sensitivity of the two techniques in detecting any additional accumulation of EB dye. Importantly, the corresponding sites to the same concentrations of the SP and CGRP mixtures show a similar trend and significant edema in Figure 1e, where analysis was carried out using the formamide extraction assay (Figure 1e). No significant edema formation was observed at any of the control sites.

### Using the formula by comparison with the formamide extraction in C57BL/6 mice

**Figure 2:**
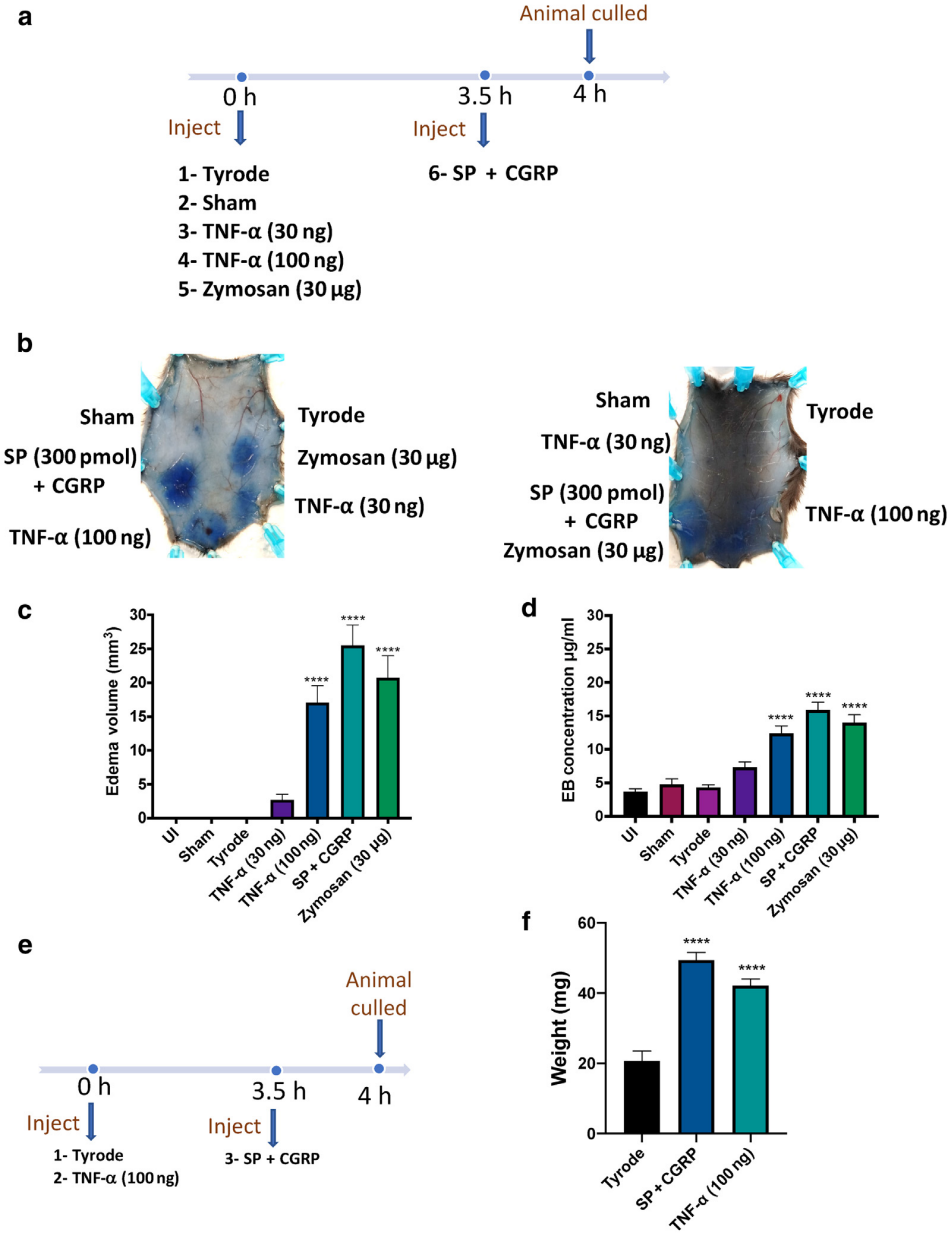
**Formamide extraction of Evans blue dye assay supports results obtained using the formula in C57BL/6 mice.** (**a**) A timeline of an experiment involving TNF-α and zymosan-induced edema protocol over 4 h, with SP + CGRP added for the last 30 minutes. Five minutes after i.v. injection of EB, i.d. injections of 50 μl of TNF-α (30 and 100 ng) and zymosan (30 μg) for 4 h, and the positive control SP (300 pmol) + CGRP for 30 minutes were injected in the presence of Tyrode, sham, and uninjected sites. (**b**) Representative results for the experiment showing a typical mouse skin (left) and a mouse with intense pigmentation in dorsal skin (right). (**c**) Results were calculated using the mathematical formula. (**d**) Results were calculated using a formamide extraction assay. Results are shown as mean ± SEM (n = 26–27). One-way ANOVA with Dunnett’s posthoc test. ∗∗∗∗*P* < 0.0001 compared with the vehicle control (Tyrode’s solution). (**e)** Timeline for an experiment involving TNF-α (100 ng, 50 μl i.d.) over 4 h, SP (300 pmol) + CGRP (20 pmol) over 30 minutes, and relevant control (Tyrode) injected 5 minutes after i.v. injection of EB. (**f**) Results determined after weighing each injected site. Results are shown as mean ± SEM (n = 5). One-way ANOVA followed by Dunnett’s posthoc test was performed. CGRP, calcitonin-gene related peptide; EB, Evans blue; h, hour; i.d., intradermal; i.v., intravenous; SP, substance P; UI, uninjected.

The next stage was to determine whether similar results were observed in C57BL/6 mice using the two different techniques. This will allow us to investigate any influence of dark skin or pigmentations on edema measurements using the formamide extraction assay and further examine the accuracy of using the formula. C57BL/6 mice were used, and in addition to the positive control of SP + CGRP for the last 30 minutes (3.5–4 hours) of the experiment, inflammatory edema was induced by injecting zymosan (30 μg i.d.) (Sigma-Aldrich, St. Louis, MO) and TNF-α (30 or 100 ng i.d.) (Novus Biological, Centennial, CO) at the start of the experiment and allowing accumulation for 4 hours (Figure 2a). TNF-α and zymosan were introduced to account for neutrophil-dependent edema formation. Zymosan (from yeast cell walls) is a general inflammatory stimulus (Rao et al., 1994). Despite some difficulties in accurately measuring edema formation in some mice with specifically dark areas of the skin (Figure 2b, right picture), these inflammatory mediators again produced significant edema and similar trends in both assays (Figure 2c and d), suggesting that the two assays can be used interchangeably. The n number in this study was chosen to allow us to include as many mice with different intensities of skin pigmentations as possible for the skin assay to ensure an accurate comparison between the skin assay and formamide extraction assay in C57BL/6 mice. Of 27 mice used in this study, only one mouse was completely pigmented, and in which edema could not be measured (as shown in Figure 2b). In some mice (4 of 26 mice), only one site of the eight edema sites injected could not be measured because of pigmentation in that particular area of the dorsal skin. Thus, complete measurements were possible in approximately 80% of the mice.

### Weight of injected sites follows a trend similar to that of measurements of edema volume

To show the importance of measuring edema volume as a marker of plasma extravasation, we carried out an experiment to weigh the injection sites after allowing edema to occur for either 30 minutes (SP + CGRP) or 4 hours (Tyrode and TNF-⍺) (Figure 2e). Our results (Figure 2f) show that the weight of each injected site depicts a response similar to that measured as edema volume and shown in Figure 2c, suggesting that edema volume is a crucial parameter to consider when estimating plasma extravasation.

### Influence of edema formation assessed through EB on the measurement of MPO

**Figure 3:**
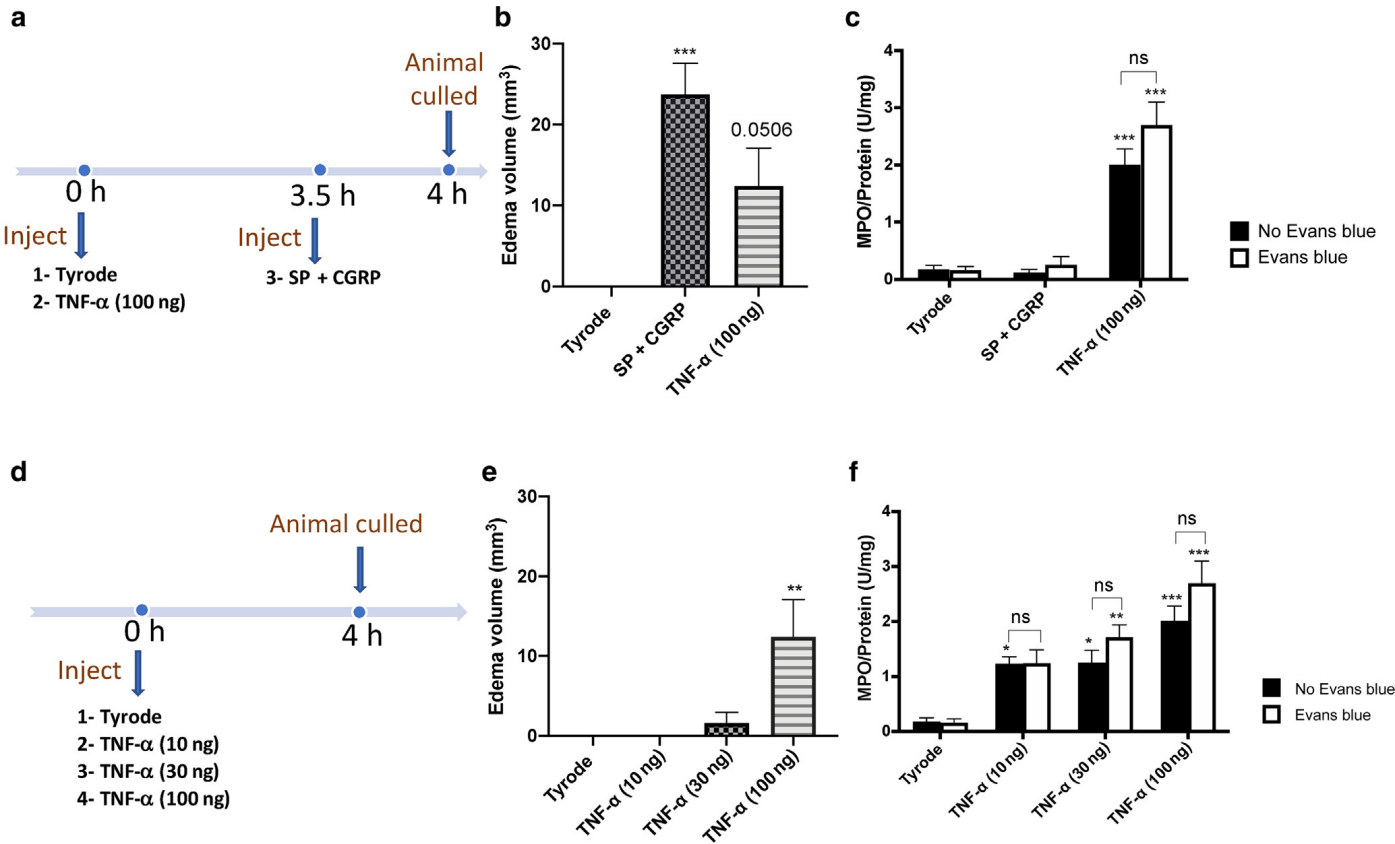
**i.v. EB dye and MPO measurements**. (**a**) Timeline of an experiment involving TNF-α (100 ng, 50 μl i.d.) over 4 h, SP (300 pmol) + CGRP (20 pmol) over 30 minutes, and relevant control (Tyrode) injected 5 minutes after i.v. injection of EB. (**b)** Results determined using the formula in **a**. (**c)** MPO activity ± EB. (**d**) Timeline of an experiment involving TNF-α (10–100 ng) over 4 h and relevant control (Tyrode) injected 5 minutes after i.v. injection of EB. Results determined using the formula in **a**. (**f**) MPO activity ± EB. Results are shown as mean ± SEM (n = 5). One-way ANOVA with Dunnett’s posthoc test or two-way ANOVA with Tukey’s posthoc test was performed. ∗*P* < 0.05, ∗∗*P* < 0.01, and ∗∗∗*P* < 0.001 compared with vehicle control (Tyrode’s solution). EB, Evans blue; h, hour; i.d., intradermal; i.v., intravenous; MPO, myeloperoxidase; ns, not significant; SP, substance P.

**Figure 4:**
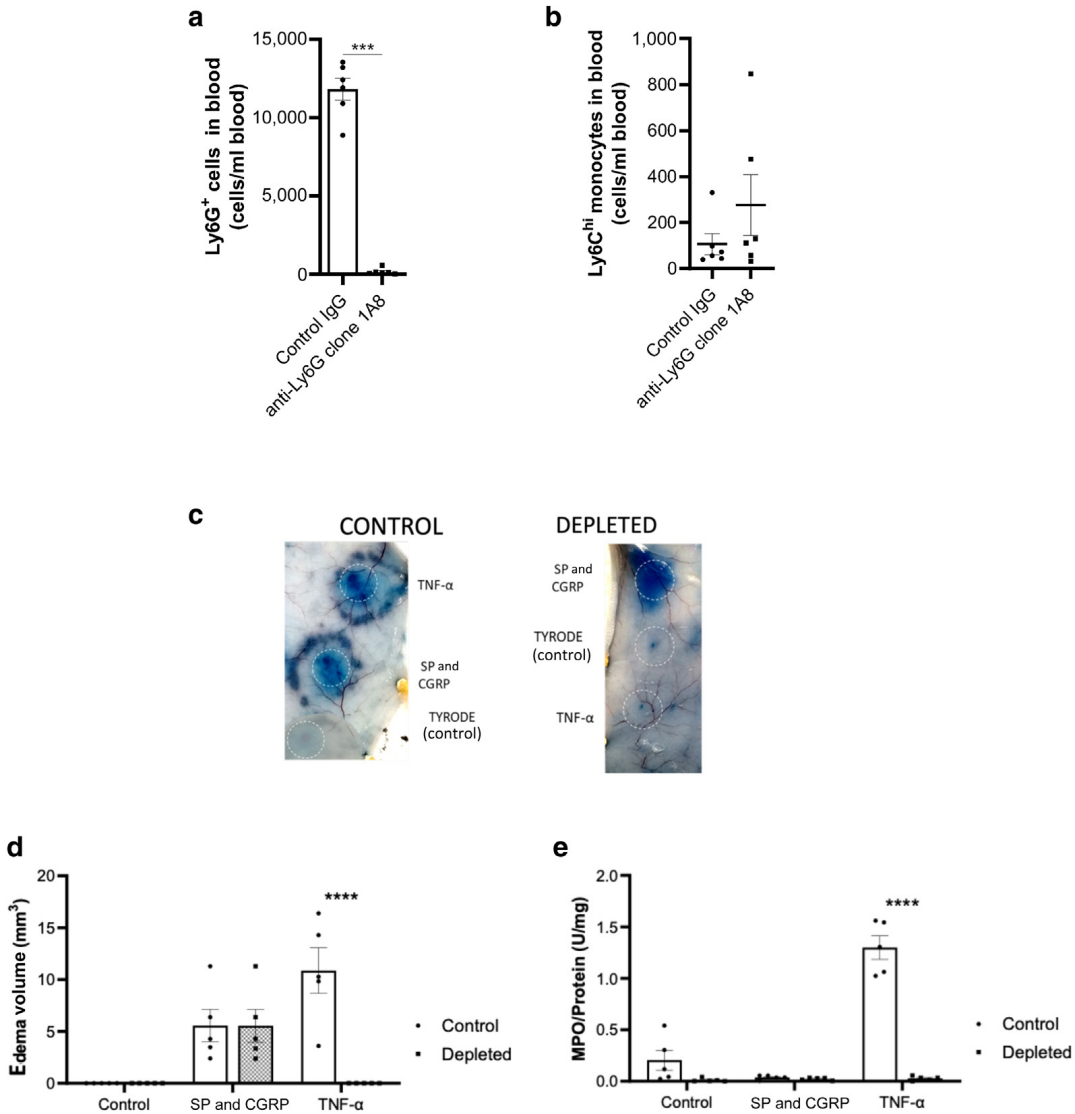
**Depletion of neutrophils reduces TNF-⍺‒induced edema and neutrophil infiltration over 4 hours.** (**a**) The number of Ly6G+ neutrophils and (**b**) Ly6C^hi^ monocytes measured in mice blood after i.p. injection with either IgG control or anti-Ly6G antibody for 24 hours. Results are shown as mean ± SEM (n = 6). *t-*test was performed. ∗∗∗*P* < 0.001 compared with control IgG. (**c**) Representative images. (**d**) Edema volume and (**e**) neutrophils infiltration measured at dorsal skin sites injected with either Tyrode and TNF-⍺ over 4 hours or SP + CGRP over 30 minutes in neutrophils-depleted mice and control mice. Results are shown as mean ± SEM (n = 5). Two-way ANOVA followed by Tukey’s posthoc test was performed. ∗∗∗∗*P* < 0.0001 compared with control mice. CGRP, calcitonin-gene related peptide; i.p., intraperitoneal; SP, substance P.

The cytokine TNF-α mediates a time-dependent edema formation after the upregulation of endothelial adhesion molecules (Xia et al., 1998). Measuring neutrophil accumulation using an MPO assay, alongside edema formation, collectively provides information on the presence of neutrophils as well as providing information on inflammatory edema formation. However, there is a problem because EB dye may affect the absorbance reading from the typical type of assay used (the 3,3′,5,5′-tetramethylbenzidine [TMB] Liquid Substrate System, Sigma-Aldrich, St. Louis, MO). To investigate this, we determined the effects of the direct-acting edema-inducing mediator SP + CGRP alongside the neutrophil-dependent edema-inducing mediator TNF-α (Figure 3a and d). The results show that significant edema formation was observed in the EB dye‒preinjected mice at SP + CGRP‒ and TNF-α‒injected sites (Figure 3b and e), with no EB dye found at the vehicle (Tyrode-injected site). The assay of MPO shows that TNF-α induced significant neutrophil accumulation over 4 hours in a manner similar in mice that previously received EB dye (i.v.) or those without EB dye but just the vehicle (saline injected, i.v.) (Figure 3c and f). In addition, no MPO was observed in the SP + CGRP site, which is expected because the edema formation is known to occur independently of neutrophils (Figure 3c). Figure 4a shows that selective neutrophil depletion is achieved with the anti-Ly6G antibody (Bio X Cell, Lebanon, NH) treatment (Daley et al., 2008), and Figure 4d shows that when neutrophils are depleted, there is an absence of edema formation in response to TNF-α but not in response to the direct-acting mediator SP + CGRP.

### Depletion of neutrophils inhibits edema formation and neutrophil infiltration induced by TNF-⍺ over 4 hours

**Figure 5:**
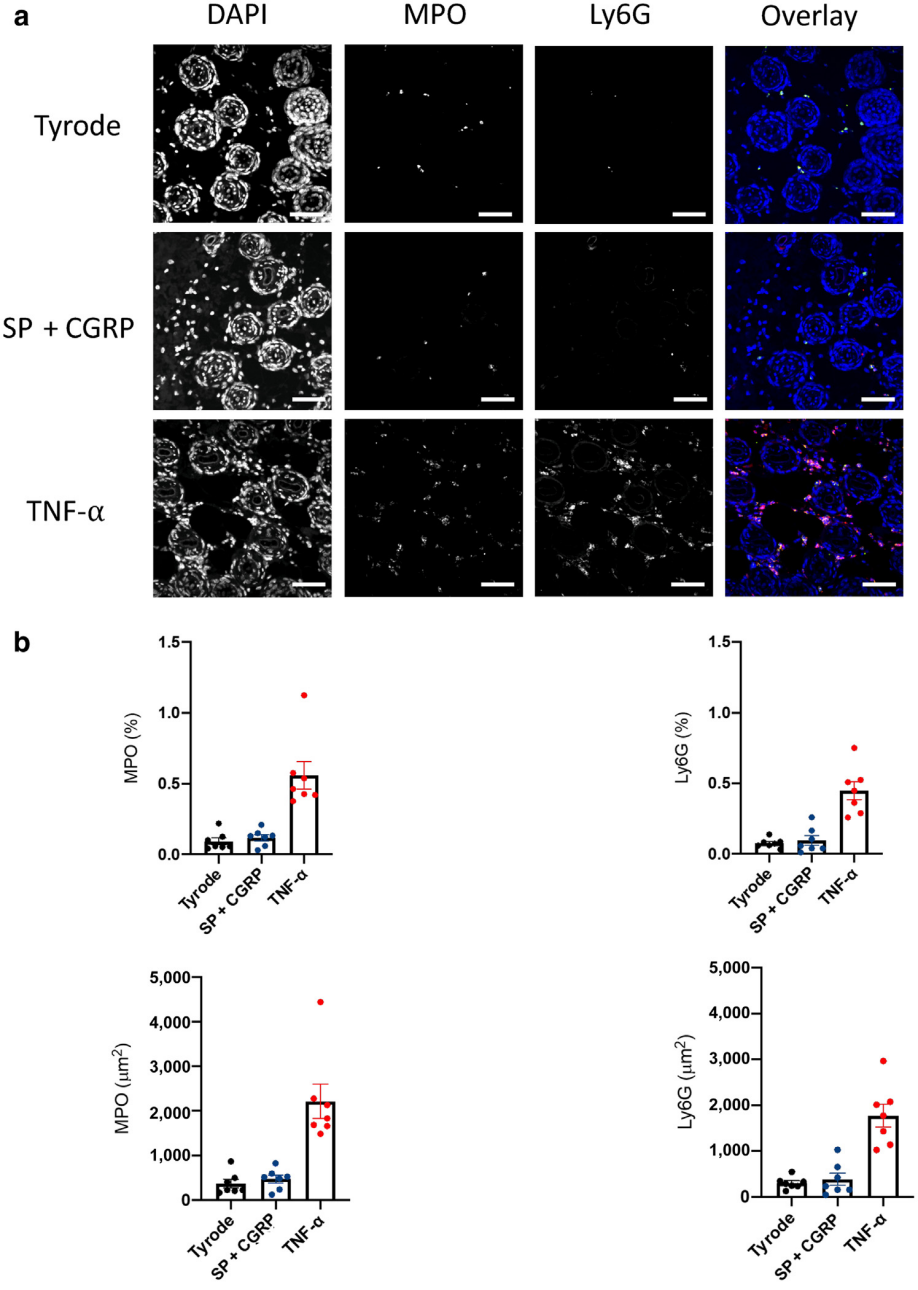
**Fluorescent images show infiltration of neutrophils at TNF-⍺‒injected site.** (**a**) Immunofluorescence representative images of neutrophil infiltration at dorsal skin sites injected (i.d.) with either Tyrode and TNF-⍺ (100 ng) over 4 hours or SP (300 pmol) + CGRP (20 pmol) over 30 minutes without injection of EB and stained with DAPI, MPO, and Ly6G antibodies. The right-hand column shows an overlay of MPO (green) and Ly6G (red) clearly showing that the majority of the MPO signal is neutrophil derived (bars = 50 μm). (**b**). Quantification of neutrophil infiltration, as assessed by immunohistochemistry, at dorsal skin sites injected with either Tyrode and TNF-⍺ (100 ng) over 4 hours or SP (300 pmol) + CGRP (20 pmol) over 30 minutes and stained with MPO and Ly6G antibodies. The results are shown for seven replicate slides from one skin sample. CGRP, calcitonin-gene related peptide; EB, Evans blue; i.d., intradermally; MPO, myeloperoxidase; SP, substance P.

To investigate the importance of neutrophils in TNF-⍺‒induced inflammation rather than other cell types in this response, we carried out an experiment where we depleted neutrophils with a specific antibody (1A8 monoclonal anti-Ly6G antibody) and studied its effect on edema and neutrophil infiltration induced by TNF-⍺ over 4 hours. Our results show successful depletion of neutrophils (Figure 4a) with no effect on monocytes (Figure 4b) in blood circulation. Moreover, we show that in neutrophil-depleted mice, TNF-⍺ only induced minimum edema (Figure 4c and d) or neutrophil infiltration (Figure 5e) over 4 hours, suggesting that neutrophils are the main cell types in this response.

### Demonstration of neutrophil infiltration at skin sites of mice not injected with EB

To further support our previous results with the MPO assay, in this study, we show neutrophil infiltration through immunohistochemistry where naïve mouse was injected (i.d.) at three different sites with either Tyrode and TNF-⍺ over 4 hours or SP + CGRP over 30 minutes and immunohistochemistry performed using MPO and Ly6G antibodies. As shown in Figure 5a, both controls (Tyrode and SP + CGRP) induce minimum neutrophil infiltration at the injection sites. However, in the site where the neutrophil chemoattractant TNF-⍺ was injected, profound neutrophil infiltration was induced (Figure 5a). Figure 5b shows an approximate estimation of the number of neutrophils at each site on the basis of two neutrophil markers used (MPO and Ly6G).

### Evaluation of the effect of EB (intradermal) on the MPO assay

**Figure 6:**
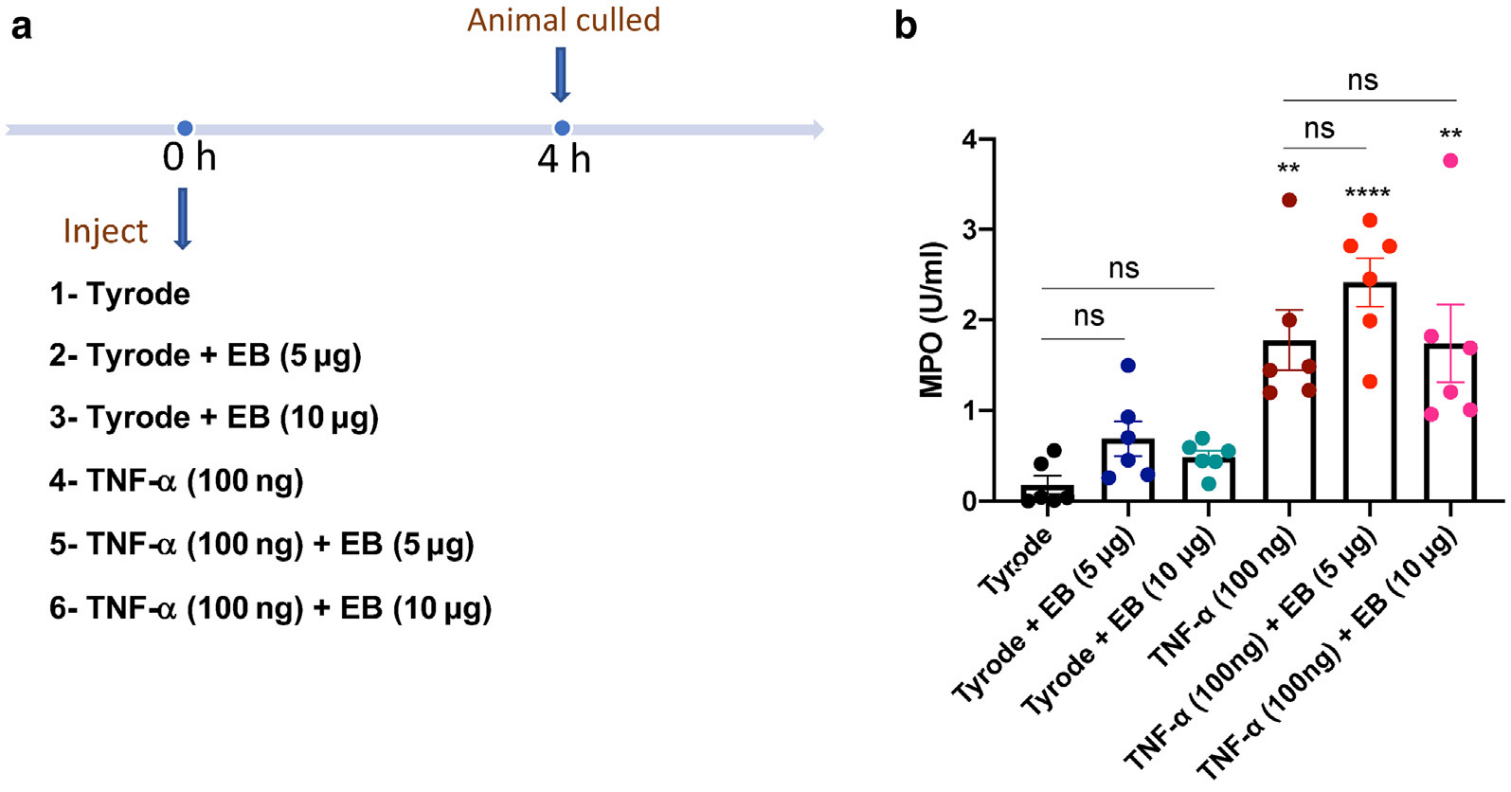
**Lack of influence of EB dye on measurement of MPO activity in skin sites**. (**a**) Shows a timeline of an experiment where two doses of EB dye (5 and 10 μg/ml) were injected (50 μl i.d.) at 0 minutes into mouse dorsal skin. Other sites were vehicle (Tyrode’s solution) and TNF-⍺ (100 ng/50 μl) ± EB treated. (**b**) MPO results are from n = 6, presented as mean ± SEM. One-way ANOVA with Tukey’s posthoc test was performed. ∗∗*P* < 0.01 and ∗∗∗∗*P* < 0.0001 compared with Tyrode treatment. EB, Evans blue; h, hour; i.d., intradermally; MPO, myeloperoxidase; ns, not significant.

To further investigate the possible influences of EB dye on the MPO assay, an experiment was designed where two concentrations of EB dye (5 and 10 μg/ml) were directly injected into the dorsal skin with either Tyrode or TNF-⍺ (Figure 6a). The doses of EB dye were chosen on the basis of the concentrations of EB dye observed after formamide extraction of skin sites (see Figure 1e). The TMB assay was then used to compare the MPO activity from EB dye‒injected sites compared with that of vehicle (Tyrode’s injected site) or TNF-⍺‒injected site alone. Figure 6b shows MPO activity induced by both treatments as expected, with no significant effect of EB on Tyrode- or TNF-⍺‒induced MPO activity.

### Effect of EB dye on MPO in vitro

**Figure 7:**
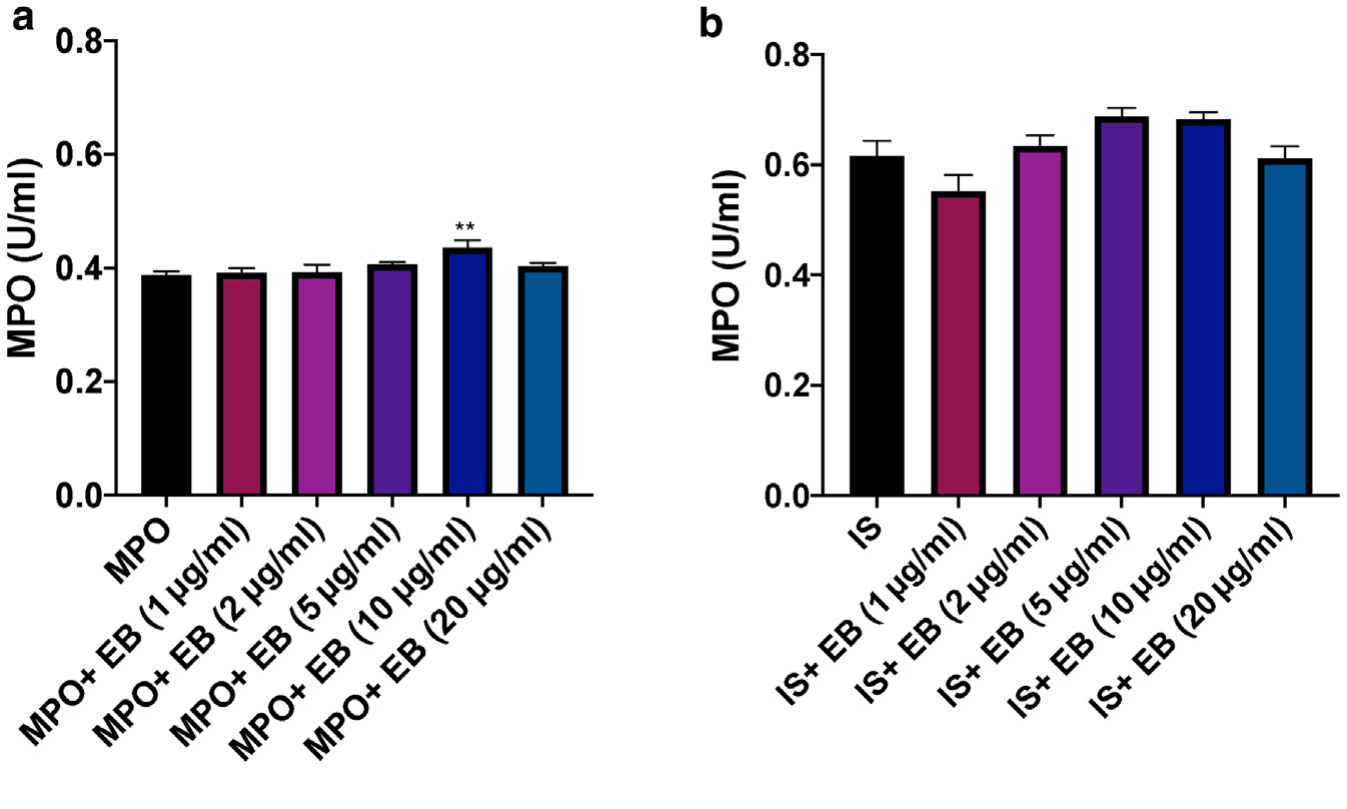
**Addition of EB dye does not significantly influence MPO activity in vitro.** (**a**) Shows the effect of increasing doses of EB dye (1, 2, 5, 10, and 20 μg/ml) on MPO activity induced by MPO enzyme (0.5 U/ml). (**b**) Shows the effect of increasing doses of EB dye (1, 2, 5, 10, and 20 μg/ml) on MPO activity induced by IS. The IS was (0.03 mg/ml zymosan) used as a positive control. Results are shown as mean ± SEM (n = 5). One-way ANOVA with Dunnett’s posthoc test was performed. ∗∗*P* < 0.01 compared with MPO enzyme. EB, Evans blue; IS, internal standard; MPO, myeloperoxidase.

We then examined the effect of different concentrations of EB dye (1–20 μg/ml) on MPO standard enzyme (0.5 U/ml) and an internal mouse MPO standard (obtained from zymosan-induced peritoneal exudate; 0.03 mg/ml). These doses of EB dye were chosen on the basis of the range of concentrations of EB dye observed through formamide extraction in Figures 1e and 2d. The concentrations of the MPO standard enzyme and the internal standard were chosen because they represent the half-maximal effective concentration of both standards. Figure 7a and b shows a similar trend of EB dye on MPO activity induced by MPO enzyme and internal standard, respectively.

### Effect of EB dye on MPO standard curve

**Figure 8:**
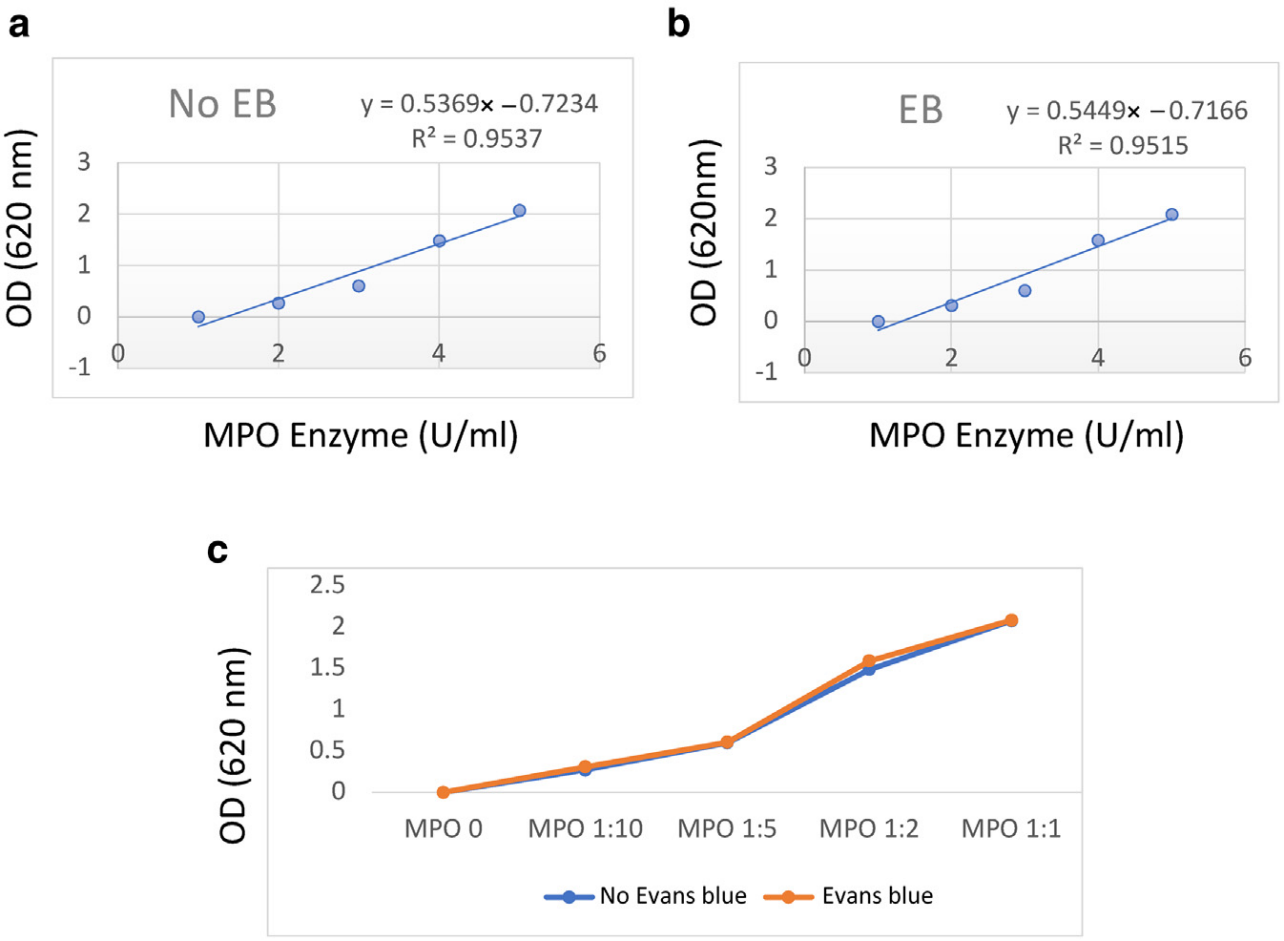
**Addition of EB dye does not affect the MPO standard curve in vitro.** (**a**) Shows the OD measurements of MPO standards (0, 0.5, 1, 1.67, and 2.5 U/ml) in the absence of EB dye. (**b)** Measurements of MPO standards (0, 0.5, 1, 1.67, and 2.5 U/ml) in the presence of EB dye (5 μg/ml), added to each sample. (**c**) Shows the results when combined in the same graph to allow for direct comparison. Absorbance was read at 620 nm. EB, Evans blue; MPO, myeloperoxidase; OD, optical density.

Finally, we carried out an experiment where EB dye (5 μg/ml) was added to each standard sample of MPO to investigate whether the dye can affect the standard curve. This dose of EB dye was chosen because it was the maximum concentration of edema formation induced by the SP + CGRP intradermal injection combination in the formamide extraction experiment in Figure 1e. As shown in Figure 8, EB dye does not cause a shift in the MPO standard curve or its coefficient of determination both when curves are shown seperately (Figure 8a and b) or combined (Figure 8c).

## Discussion

The results show that the use of an ellipsoidal-based mathematical formula is capable of estimating edema volume in mouse skin. The analysis is further validated by similar results to those obtained by formamide extraction. It provides an alternative approach to measuring edema formation, except for mice with heavily pigmented skin. Furthermore, the results indicate that in the amounts that accumulate in the skin during inflammatory edema formation, EB dye does not significantly influence MPO activity, thus allowing edema formation and neutrophil accumulation to be determined in the same skin site. This leads to a potential 50% reduction in the mouse numbers required, in keeping with 3R (replacement, reduction, and refinement) considerations. Although the importance of the role of inflammatory swelling is sometimes debated, it remains a cardinal sign of inflammation, the regulation of which remains under study.

Edema formation is the result of plasma extravasation that follows an increase in microvascular permeability that is not removed by the time of lymphatic clearance (Park-Windhol and D'Amore, 2016). A major goal was to test the ability of the formula for determining ellipsoid volume as a quantitative estimate of edema formation. Although edema measurement based on the width of the wheal gives an indication of the spread of the edema formed (Miles and Miles, 1952), calculating edema volume provides a more conclusive estimation because it takes into account the depth as well as the spread of the wheal. Moreover, measuring edema volume is more clinically relevant because volume-based methods with reliable results such as the water displacement technique have been used in both preclinical and clinical settings to estimate foot and ankle volume (Brodovicz et al., 2009). Furthermore, this specific equation has been used successfully to measure prostate volume in patients with benign prostatic hyperplasia (Eri et al., 2002). Although understanding that computer-assisted techniques may be considered valuable, in this case, their use is limited. They are usually more expensive and time consuming and not sensitive enough for measuring multiple sites in quick succession. This makes it difficult to analyze and harvest tissue from multiple sites simultaneously. Such techniques (e.g., involving ultrasound) can be used to estimate skin tumor size (Leung et al., 2013).

One limitation of using the equation is that it can only be used when visualization of the edema site is possible. In certain dark-skinned mice where strong intensity of dorsal pigmentation is present (Figure 2b), the analysis for the mathematical formula cannot be performed. Fortunately, on the basis of our results, this appears to be a rare problem because only 1 mouse of over 27 mice has completely pigmented skin, which made measuring edema impossible. A few other mice (four) had partially pigmented skin affecting measurement at one site.

In this situation, the classic, more time-consuming, measurement of edema formation based on EB dye concentration through a formamide extraction assay may have to be used. However, this is not compatible with many other assays because the bioactivity of the enzymes and proteins is disrupted during the extraction process. By comparison, using the mathematical formula to measure edema volume leaves the skin sites intact for further analysis of the same samples (e.g., in the MPO assay).

The measurement of the MPO content in the dorsal skin allows for assessment of neutrophil accumulation (Bradley et al., 1982). We show a significant increase in edema formation and neutrophil accumulation in the TNF-α‒treated sites within 4 hours of injection. In contrast, no significant MPO readout but significant edema formation was achieved with the neutrophil-independent edema formation mixture of SP + CGRP over 30 minutes, which stimulates a neutrophil-independent acute response. The measurement of neutrophils alongside edema formation provides a powerful mechanistic tool for a neutrophil-dependent inflammatory mechanism as well as for the study of potential drugs that influence neutrophil migration. The possibility of measuring edema formation and neutrophil accumulation in the same tissue has been previously investigated, but the studies either showed conflicting and inconclusive results or lacked positive controls (Fliss and Gattinger, 1996; Griswold et al., 1989; Mullane et al., 1985).

Further experiments in this study were designed to examine the extent to which EB dye may interfere with MPO measurements. First, MPO activity was compared between two groups that were preinjected (i.v.) with either EB dye or vehicle (saline). No significant differences were found in MPO activity, although a trend toward an increase in MPO activity was seen in the EB-preinjected mice, as also noted by Griswold et al. (1989) when attempting a similar correlation. Second, the effect of EB dye on the skin was probed by injecting EB dye i.d. to maximize the chance of picking up any interference. Again, no significant difference was found in MPO activity in Tyrode-treated or TNF-⍺‒treated sites in the presence or absence of EB dye. Third, in in vitro experiments, a series of EB dye concentrations were added to a fixed concentration of MPO. Overall, little effect of EB dye with MPO activity was observed, when taking into account all the doses tested. Finally, we spiked an MPO standard curve with EB dye at a dose chosen to represent the EB concentration induced by SP + CGRP (Figure 1e). This allowed investigation for a shift to the left of the curve by the presence of the EB dye. Again, no significant change was observed in the slope or the linearity of the standard curve of MPO activity.

In conclusion, we propose the use of the prolate ellipsoid formula to measure edema volume in mouse dorsal skin to complement the widely used formamide extraction assay. The mathematical formula can be used in all but heavily pigmented mouse skin. The similarity in measurements between the two different techniques indicates that there is no adverse bias in either of the measurement techniques. Furthermore, data from this study extend previous studies to provide strong evidence that EB dye does not significantly interfere with MPO measurements. Consequently, we propose that edema volume based on mathematical formula and neutrophil accumulation can be measured in the same skin site, which will reduce the number of mice used and minimize interanimal variability.

## Materials and Methods

### Mice

Male CD1 and male and female C57BL/6 mice (aged 8–12 weeks, Charles River Laboratories, Currie, United Kingdom) were acclimatized in a climatically controlled environment and allowed food and water ad libitum. All experiments were carried out in accordance with the UK Home Office Animals (Scientific Procedures) Act 1986, with ethics approval by King’s College Animal Care and Ethics Committee. The study was carried out in compliance with the ARRIVE (Animal Research: Reporting of In Vivo Experiments) guidelines.

### The skin assay to measure edema volume

Mice were weighed and anesthetized with 2% isoflurane (Isocare, Animalcare, York, United Kingdom) carried in 1L/min oxygen and i.v. injected with EB dye (1.25% w/v in saline, 0.1 mL/mouse, Sigma-Aldrich) after shaving the dorsal skin. Sites were marked out according to a balanced site design for intradermal injections (50 μl) using a 27G needle. After randomly assigning agents, the skin was injected with SP (Sigma-Aldrich, St. Louis, MO) (300 or 500 pmol), CGRP (AnaSpec, Fremont, CA) (20 pmol), TNF-α (Novus Biological, Centennial, CO) (10, 30, and 100 ng), and/or zymosan (Sigma-Aldrich, St. Louis, MO) (30 μg) and vehicles, depending on the instructions. Agents were dissolved in Tyrode’s solution (50 μl Tyrode’s solution: 137 mM sodium chloride, 2.68 mM potassium chloride, 0.4 mM sodium dihydrogen phosphate, 11.9 mM sodium bicarbonate, 0.5 mM magnesium chloride, and 5.6 mM glucose in distilled water, all reagents are from Sigma-Aldrich, St. Louis, MO) used as the vehicle control for intradermal injections. Uninjected and sham-injected (sites that received injection needle prick but no fluid) sites were also investigated. Mice were allowed to recover when necessary, and at the predesignated time, they were reanesthetized (if required) and killed by cervical dislocation. Dorsal skin was carefully blunt dissected, collected, and pinned nonfur side up without excessive pulling to allow clearer visualization of the edema bleb. Two perpendicular diameters (length and width) of the edema formed were measured in millimeters using a ruler, and the edema depth was measured in millimeters by placing the edema site within an engineering caliper (Moore and Wright, Sheffield, United Kingdom). The caliper was adjusted until it touched but did not squeeze both surfaces of the skin area. The caliper was then locked to ensure that the measurement remained while removing it from the skin. The three diameters (length, width, and depth) of the ellipsoid-shaped edema were then inputted into the following equation to calculate edema volume (Figure 1a): Edema volume (mm³) = (π/6) × length × width × depth.

### Formamide extraction of EB dye

Skin sites were punched out (10 mm diameter punch), immediately placed in 450 μl formamide (Sigma-Aldrich, St. Louis, MO), and vortexed. After incubation (24 hours at 56 °C), samples were vortexed and centrifuged (1 minute at 17,000 g). Aliquots (100 μl per sample) were transferred into a 96-well plate in duplicates, and a standard curve of EB dye diluted in formamide (0‒25 μg/ml) was plotted to enable the determination of the EB dye absolute concentration. Absorbance (OD) was read at 620 nm.

### MPO assay

MPO assay was adapted from the study by Bradley et al. (1982). Skin sites were prepared as described earlier, homogenized in 500 μl homogenization buffer (0.1 M sodium chloride, 0.02 M sodium phosphate, 0.015 M EDTA, 0.5% hexadecyl trimethyl ammonium bromide, pH 4.7 , all reagents are from Sigma-Aldrich, St. Louis, MO), and lyzed using QIAGEN TissueLyser II (Thermo Fisher Scientific, Warrington, United Kingdom) at 30 Hz for 5 minutes, three times, cooling between. Samples were centrifuged at 17,000*g* for 15 minutes at 4 °C, and the supernatant was collected. Reactions were performed in a 96-well plate at room temperature. Hydrogen peroxide oxidation of TMB Liquid Substrate System (TMB, Sigma-Aldrich, St. Louis, MO) was used to determine MPO activity. In a 96-well plate, 25 μl of MPO buffer was added to 25 μl of the sample, and 100 μl of TMB liquid substrate was then added. The plate was incubated in the dark at 37 °C for 15 minutes. Absorbance (OD) was read at 620 nm using a spectrophotometer, and a standard curve of OD against MPO in the standard samples was plotted. In some cases, protein (BSA) concentrations were also measured by adding 25 μl of alkaline copper tartrate solution (Bio-Rad Laboratories, Watford, United Kingdom) to 1 μl of the sample (diluted 1:5 in homogenization buffer), and 200 μl of dilute Folin (Bio-Rad Laboratories, Watford, United Kingdom) was then added. OD was read at 700 nm, thus acquiring the quantification of MPO/protein in each condition.

### Obtaining an internal standard through Zymosan-induced peritoneal inflammation

This procedure was adapted from the study by Cao et al. (2000). To develop a reference sample of MPO, a sample was prepared from mouse peritoneal inflammatory exudate. The exudate was obtained by inducing peritoneal inflammation by zymosan (1 mg/0.5 ml intraperitoneally) for 4 hours.

### Neutrophils depletion and FACS analysis

Neutrophil depletion was achieved by intraperitoneal doses of 1A8 monoclonal anti-Ly6G antibody (Bio X Cell, Lebanon, NH) at 25 mg/kg in saline. Equivalent doses of IgG monoclonal anti-trinitrophenol (clone 2A3, Bio X Cell, Lebanon, NH) were administered to control mice 24 hours before carrying out the dorsal skin edema inflammation model. Four hours after dorsal skin model, mice were killed by anaesthetization, and whole blood was collected through cardiac puncture for flow cytometric analysis of neutrophils. The heparinized blood (1:100, stock heparin 5,000 IU/ml) was incubated with the following antibodies: anti-Ly6G-BV785 (1:200, catalog 127645, BioLegend, San Diego, CA) and anti-Ly6C BV605 (1:200, catalog 128035, BioLegend, San Diego, CA). Samples were acquired in an LSRFORTESSA flow cytometer (BD Biosciences, Oxford, United Kingdom) and analyzed using FlowJo software 9.7.5 (FlowJo, Ashland, OR).

### Immunohistochemistry

Skin samples were fixed in 4% paraformaldehyde (overnight, 4 °C). After fixation, they were embedded in paraffin wax. Approximately 8‒10 transverse sections were obtained from each skin site (5 μm) using a MICROM HEIDELBERG model HM330 microtome (Thermo Fisher Scientific, Warrington, United Kingdom) for staining. Antigen retrieval was achieved using Tris-EDTA buffer (10 mM Tris Base [T1503, Sigma-Aldrich], 1 mM EDTA [EDS, Sigma-Aldrich, St. Louis, MO] solution, pH 9.0), and slides were permeabilized with 0.1‒0.2% Triton X-100 (X100, Sigma-Aldrich, St. Louis, MO) at 4 °C. Primary antibodies used are Ly6G rabbit monoclonal E6Z1T (87048, Cell Signaling Technology, Danvers, MA) at 1:100 and MPO goat polyclonal (AF3667, R & D Systems, Minneapolis, MN) at 1:200. Secondary antibodies used are donkey anti-rabbit IgG secondary antibody (1:250, A10040, Invitrogen Alexa Fluor 546, Thermo Fisher Scientific, Warrington, United Kingdom) and donkey anti-goat IgG secondary antibody (1:250, A-11055, Invitrogen Alexa Fluor 488, Thermo Fisher Scientific, Warrington, United Kingdom). Images were captured with confocal microscopy (A1R HD, Nikon, Tokyo, Japan) at ×20 magnification and analyzed using ImageJ software (National Institutes of Health, Bethesda, MD). (bars = 50 μm).

### Statistical analysis

Results are shown as mean ± SEM and analyzed by *t*-test or one-way and two-way ANOVA followed by Dunnett’s or Tukey’s posthoc tests. Statistics were performed using GraphPad Prism 8 software. A probability level ≤0.05 was used to indicate statistical significance.

### Data availability statement

No datasets were generated or analyzed during this study.

## ORCIDs

Ali A. Zarban: http://orcid.org/0000-0003-0328-5032

Hiba Chaudhry: http://orcid.org/0000-0002-8549-215x

Davide Maselli: http://orcid.org/0000-0002-2193-1678

Xenia Kodji: http://orcid.org/0000-0002-5822-685X

Joao de Sousa Valente: http://orcid.org/0000-0003-1888-6324

Justin Joachim: http://orcid.org/0000-0002-3748-6900

Silvia Cellone Trevelin: http://orcid.org/0000-0003-2722-2458

Johannes van Baardewijk: http://orcid.org/0000-0002-7515-3162

Fulye Argunhan: http://orcid.org/0000-0001-7616-2151

Alexandar Ivetic: http://orcid.org/0000-0001-9844-6518

Manasi Nandi: http://orcid.org/0000-0001-8585-5363

Susan D. Brain: http://orcid.org/0000-0002-9684-8342

## Conflict of Interest

The authors declare no conflict of interest.
